# Residual mitochondrial transmembrane potential decreases unsaturated fatty acid level in sake yeast during alcoholic fermentation

**DOI:** 10.7717/peerj.1552

**Published:** 2016-01-14

**Authors:** Kazutaka Sawada, Hiroshi Kitagaki

**Affiliations:** 1 Department of Biochemistry and Applied Biosciences, United Graduate School of Agricultural Sciences, Kagoshima University, Kagoshima, Japan; 2 Industrial Technology Center of Saga Prefecture, Saga city, Saga, Japan; 3 Department of Biochemistry and Applied Biosciences, United Graduate School of Agricultural Sciences, Kagoshima University, Japan; 4 Department of Environmental Science, Faculty of Agriculture, Saga University, Saga city, Saga, Japan

**Keywords:** Sake yeast, Mitochondria, Unsaturated fatty acid, Alcoholic fermentation, Oxygen, Anaerobiosis

## Abstract

Oxygen, a key nutrient in alcoholic fermentation, is rapidly depleted during this process. Several pathways of oxygen utilization have been reported in the yeast *Saccharomyces cerevisiae* during alcoholic fermentation, namely synthesis of unsaturated fatty acid, sterols and heme, and the mitochondrial electron transport chain. However, the interaction between these pathways has not been investigated. In this study, we showed that the major proportion of unsaturated fatty acids of ester-linked lipids in sake fermentation mash is derived from the sake yeast rather than from rice or koji (rice fermented with *Aspergillus*). Additionally, during alcoholic fermentation, inhibition of the residual mitochondrial activity of sake yeast increases the levels of unsaturated fatty acids of ester-linked lipids. These findings indicate that the residual activity of the mitochondrial electron transport chain reduces molecular oxygen levels and decreases the synthesis of unsaturated fatty acids, thereby increasing the synthesis of estery flavors by sake yeast. This is the first report of a novel link between residual mitochondrial transmembrane potential and the synthesis of unsaturated fatty acids by the brewery yeast during alcoholic fermentation.

## Introduction

Sake, the traditional rice wine of Japan, has a history of more than 1,100 years (Kitagaki & Kitamoto, 2013). Sake is manufactured by mixing koji (rice fermented with *Aspergillus,* which catalyzes the breakdown of starch), steamed rice, water, and the sake yeast *Saccharomyces cerevisiae*, and brewing the mash for 20–30 days. Sake is brewed at 12–20 °C and thus, sake yeast grows well at these temperatures as compared to other yeasts. However, sake yeast also grows well at higher temperatures (30 °C), both aerobically and anaerobically. Refined sake, which contains abundant estery flavors such as those of isoamylacetate and ethylcaproate, is preferred worldwide. The estery flavors in sake are produced during fermentation by sake yeasts. The augmentation of estery flavors by sake yeasts is key for the production of high quality sake (Kitagaki & Kitamoto, 2013). During sake brewing, oxygen is rapidly depleted within 1–2 days due to the consumption of oxygen and production of CO_2_ by sake yeasts (Nagai et al., 1992); however, a limited amount of oxygen may be added by stirring the fermentation mash with a rod at 1–2 day intervals.

Lipid components, such as unsaturated fatty acids (Fujii et al., 1997), sterols (Ohta & Hayashida, 1983), and glucosylceramide (Sawada et al., 2015) affect the fermentation profiles of yeast. In particular, the presence of unsaturated fatty acid results in a decrease in the production of isoamylacetate by brewery yeasts via repression of the alcohol acetyltransferase gene *ATF1* (Yoshimoto et al., 1998; Fujii et al., 1997; Fujiwara et al., 1998). Therefore, unsaturated fatty acid content is targeted to control isoamylacetate production by sake yeasts. Additionally, unsaturated fatty acids regulate ethanol tolerance (You, Rosenfield & Knipple, 2003). To date, however, efforts to regulate the synthesis of unsaturated fatty acid have been focused solely on molecular oxygen content (Fujii et al., 1997; Nakagawa, Sugioka & Kaneko, 2001) and acyltransferase activity (De Smet et al., 2012). Alternative factors that potentially regulate the content of unsaturated fatty acids in sake yeast remain unknown.

Oxygen is required for various biosynthetic pathways of yeast, including those involved in the synthesis of unsaturated fatty acids (Mitchell & Martin, 1995), sterols (Fornairon-Bonnefond et al., 2003), heme synthesis (Maines, 1988), oxidation of lipids by reactive oxygen radicals (Salmon et al., 2000), cell wall protein expression (Kitagaki, Shimoi & Itoh, 1997), and the expression of diauxic shift-related genes (Kitagaki et al., 2009). However, oxygen is depleted in the very early phase of alcoholic fermentation. As a result, the availability of molecular oxygen is limited during alcoholic fermentation. The utilization of oxygen during alcoholic fermentation via 2 major pathways, fatty acid desaturation and sterol synthesis, has been precisely investigated (Rosenfeld & Beauvoit, 2003a; Rosenfeld et al., 2003b). In addition to these pathways, the mitochondrial electron transport chain which utilizes molecular oxygen (O’Connor-Cox, Lodolo & Axcell, 1996) and nonclassical mitochondrial electron transport chain activity which produces nitric oxide from ${\[{\rm{NO}}_2^ - \]}$ (Castello et al., 2008) have been reported. However, there are few reports on the interactions of the mitochondrial electron transport chain and other pathways during alcoholic fermentation.

In previous studies, we have demonstrated that mitochondrial activities, morphologies or degradation of sake yeast affect fermentation characteristics such as malic acid, pyruvic acid productivity and carbon flux (Kitagaki et al., 2008; Kitagaki, 2009; Horie et al., 2010; Motomura, Horie & Kitagaki, 2012; Shiroma et al., 2014; Kitagaki & Takagi, 2014; Agrimi et al., 2014; Oba et al., 2014). Based on these studies, we hypothesize that the residual mitochondrial electron transport chain activity of brewery yeasts is the determinant of unsaturated fatty acid production efficiency.

In the present study, we show that the major proportion of fatty acids which are ester-linked to glycerophospholipids and neutral lipids in the fermentation mash is derived from sake yeast, not rice or koji, and the synthesis of the unsaturated fatty acids in sake yeast increases when the activity of the mitochondrial electron transport chain is inhibited. To our knowledge, this is the first report indicating that residual mitochondrial activity is essential for regulating the content of unsaturated fatty acids in fermentation mash, providing a valuable insight into the relationship between mitochondrial activity and the ester-producing ability of brewery yeasts.

## Materials and Methods

### Strains and media

Sake yeast RAK1536 K7 *his3/his3* + pRS413-GPDmitoGFP (Kitagaki et al., 2008; Hashimoto et al., 2005) and laboratory yeast CEN.PK2 + pRS413-GPDmit obtained from Euroscarf (Entian & Kotter, 1998) were used in this study. For culturing of these yeasts, CSM (-HIS) medium (0.67% Difco^tm^ Yeast Nitrogen Base w/o Amino Acids and Ammonium Sulfate, 0.08% Complete Supplement Mixture Drop-out: -HIS, and 2% glucose) was used.

### Analysis of unsaturated fatty acid level

In order to analyze the amount of fatty acids ester-linked to glycerophospholipids and neutral lipids in the fermentation mash, 30 μl of 0.2 mg/ml heptadecanoic acid was added to the extracted solution as an internal control.

For preparation of the fermentation mash, 12.6 g pregelatinized rice (Tokushima seiko, Co. Ltd., Awa, Japan) with 30% of its surface polished and removed, 4.8 g pregelatinized koji (Tokushima seiko, Co. Ltd., Awa, Japan) with 30% of the surface of rice polished and removed, and 42 ml distilled water were mixed. In order to prepare the fermentation mash with yeast, yeast was added to the mash at 1 × 10^7^ cells/ml and incubated at 30 °C for 7 days. For preparation of the fermentation mash without yeast, the mash was directly frozen without adding yeast. The mash was freeze-dried and 20 mg, 80 mg, or 320 mg of the freeze-dried samples were subjected to fatty acid analysis. For analysis of the fatty acid composition ester-linked to glycerophospholipids and neutral lipids of yeast cells, cells were collected after incubation by centrifugation at 16,900 g for 1 min, washed twice with distilled water, and freeze-dried. An aliquot (20 mg) of the sample was subjected to fatty acid analysis. Degradation of the ester bond of lipids, methylation of the fatty acids and purification by silica gel chromatography was performed according to the manufacturer’s protocol. The derivatized fatty acids were analyzed by gas chromatography (Shimadzu GC-2014; initial temperature 240 °C, hold 5 min, 4 °C/min, maximum 240 °C, hold 5 min, sampling time 1 min, nitrogen gas, pressure 85.3 kPa) equipped with a DB-WAX column (length 30.0 m, 0.25 mm ID, film thickness 0.25 μm), an AOC-20 autosampler (sampling volume 1 μl, split ratio 1:50), and Supelco FAME standard (Sigma Aldrichh, St. Louis, MO, USA) as a reference.

### Analysis of yeast physiology following oxygen shutoff

Yeast cells (K7 *his3/his3* RAK1536 + pRS413-GPDmitoGFP and CEN.PK2+pRS413-GPDmit) were precultured in selective medium, inoculated into 1 ml of CSM medium at 1.0 × 10^6^ cells/ml with a layer of liquid paraffin on top of the culture medium, and incubated statically at 30 °C for 4 h, 12 h, and 24 h, with or without 0.004% w/v resalurin (Sigma-Aldrichh, St. Louis, MO, USA). Formaldehyde (3.7% v/v) was added and the culture was incubated at room temperature for 30 min. Cells were collected by centrifugation, washed twice with distilled water, and visualized under a fluorescent microscope.

### Statistical analysis

Fermentation experiments were performed in triplicate from independent starter cultures. Significant differences between the averages of the data were calculated by unpaired two-tailed Student’s *t*-test.

## Results

### Sake yeast is the main source of unsaturated fatty acids in fermentation mash

First, in order to elucidate the source of unsaturated fatty acids in the fermentation mash, their composition was investigated. As koji (catalyst of starch), rice pre-fermented with *A. oryzae,* rice, and yeast are used for sake brewing, the same amount of koji and rice was fermented with or without yeast, and its fatty acid composition was analyzed. We found that the oleic acid composition of fermentation mash with yeast was 136-fold higher than that of fermentation mash without yeast, palmitic acid composition of fermentation mash with yeast was 137-fold higher than that of fermentation mash without yeast and polyunsaturated fatty acids such as linoleic acid and α-linolenic acid that are contained in rice and koji were not detected (Table 1). These results clearly indicate that sake yeast, which contains palmitic acid and oleic acid, but not rice or koji, which contains linoleic acid and α-linolenic acid, is the main source of unsaturated fatty acids of the sake mash.

**Table 1:** Fatty acid composition of fermentation mash with or without yeast. Fermentation mash was incubated with or without sake yeast RAK1536. Lipids extracted from the freeze-dried mash were added with the internal control heptadecanoic acid and were derivatized by fatty acid methylation. The derivatized fatty acid was applied to gas chromatography analysis. The results are mean values ± standard errors of means of two analyses.

Fatty acid composition (mg/g)	Mash with yeast	Mash without yeast
Palmitic acid	30.5 ± 0.0066	0.222 ± 0.0964
Palmitoleic acid	5.54 ± 0.327	n. d.
Stearic acid	2.89 ± 0.415	n. d.
Oleic acid	18.7 ± 3.43	0.138 ± 0.0968

### Mitochondrial morphology remains tubular after oxygen shutoff

**Figure 1:**
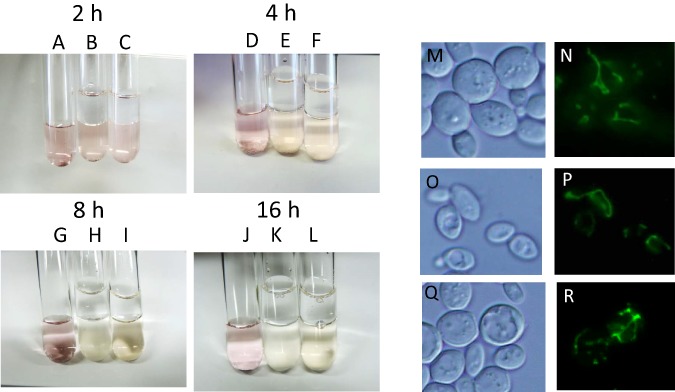
Residual mitochondrial transmembrane potential decreases unsaturated fatty acid level in sake yeast during alcoholic fermentation oxidoreductive status and mitochondrial morphology of sake yeast during alcoholic fermentation. Sake yeasts with visualized mitochondria (RAK1536 K7 *his3/his3* +pRS413-GPDmitoGFP) were incubated by shaking in selective synthetic media, inoculated into media with a layer of liquid paraffin on top, and incubated statically. (A-D) Rezarulin (0.004 % w/v) was added to the culture, and the oxidoreductive state of the culture was monitored by its color. (A) 2 h without yeast (B) 2 h with sake yeast (C) 2 h with laboratory yeast (D) 4 h without yeast (E) 4 h with sake yeast (F) 4 h with laboratory yeast (G) 8 h without yeast (H) 8 h with sake yeast (I) 8 h with laboratory yeast (J) 16 h without yeast (K) 16 h with sake yeast (L) 16 h with laboratory yeast. Sake yeast RAK1536 K7 *his3/his3* +pRS413-GPDmitoGFP and laboratory yeast CEN.PK2 + pRS413-GPDmit were used. Sake yeasts cultured under alcoholic fermentation for 4 h (M; DIC, N; GFP), 12 h (O; DIC, P; GFP), and 24 h (Q; DIC, R; GFP) were fixed with formaldehyde and observed under a fluorescent microscope. Detailed methods are described under Materials and Methods. The results shown are representative of at least two independent fermentation experiments.

During alcoholic fermentation on an industrial scale, molecular oxygen is depleted after 1–2 days of fermentation (Nagai et al., 1992) due to consumption by yeast and the production of a large quantity of CO_2_, which is heavier than air. Therefore, after 1–2 days, yeast is exposed to extreme anaerobiosis. In order to simulate industrial fermentation, we designed a culture system to deplete molecular oxygen during alcoholic fermentation. Immediately after the start of fermentation, a layer of liquid paraffin, which functions as an oxygen valve, was added on top of the culture media, and fermentation was carried out by sake yeast. The concentration of oxygen after oxygen shutoff was monitored using the oxidoreductive stain rezarulin. The color of the culture indicated that oxygen is depleted within 8 h of the start of fermentation when incubated with sake and laboratory yeasts (Figs. 1A–1L). In order to investigate the mitochondrial status of sake yeast under these conditions, the mitochondrial morphology of sake yeast was examined. Mitochondrial morphology is an indicator of mitochondrial transmembrane potential (Benard et al., 2007). It was found that most of the mitochondria of sake yeast were tubular at 4 h after oxygen shutoff, but gradually fragmented 12 h after the start of fermentation; however, a certain portion of mitochondria stayed tubular at 24 h after start of the culture (Figs. 1M–1R). This result indicates that oxygen is depleted during the early phase of alcoholic fermentation upon oxygen shutoff; however, mitochondrial electron potential is retained during anaerobic stages of alcoholic fermentation.

### Inhibition of mitochondrial electron potential elevates synthesis of unsaturated fatty acids

**Figure 2:**
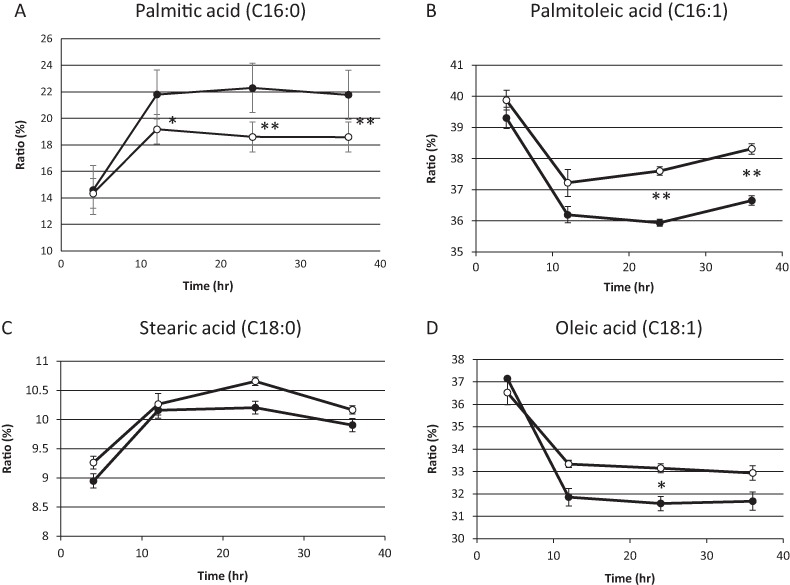
Fatty acid composition of sake yeast during alcoholic fermentation challenged with or without mitochondrial inhibitor. Sake yeast (RAK1536 K7 *his3/his3* + pRS413-GPDmitoGFP) were incubated by shaking in selective synthetic media. Yeast cells (1.0 × 10^6^ cells/ml) were inoculated into 10 ml media, with or without 20 μM carbonyl cyanide m-chlorophenylhydrazone. The culture was covered by adding a layer of liquid paraffin on top of the media, and incubated statically. Yeast cells were collected by centrifugation and lipids were extracted with chloroform/methanol. Extracted lipids were subjected to derivatization and analyzed by GC analysis. (A) Palmitic acid (B) Palmitoleic acid (C) Stearic acid (D) Oleic acid. Closed squares represent the results without 20 μM carbonyl cyanide m-chlorophenylhydrazone and open squares represent the results with 20 μM carbonyl cyanide m-chlorophenylhydrazone. Experiments were performed in triplicate from the respective starter cultures. The results are expressed as mean values ± standard errors of means. Significant differences were calculated by unpaired two-tailed Student’s *t*-test (^**^, *p* < 0.01, ^*^, *p* < 0.05). Detailed methods are described under Materials and methods.

On the basis of the above results, we hypothesized that residual mitochondrial electron transport chain activity results in the utilization of the low levels of molecular oxygen available, thereby limiting the availability of molecular oxygen for fatty acid desaturation within endoplasmic reticulum in sake yeast. In order to verify this hypothesis, the mitochondrial uncoupler, carbonyl cyanide m-chlorophenylhydrazone, was added to the medium during alcoholic fermentation, and the composition of fatty acids was monitored. We found that the level of unsaturated fatty acids (oleic acid and palmitoleic acid) were increased in response to mitochondrial uncoupler (Figs. 2B and 2D, *p* < 0.05), whereas the level of saturated fatty acid, stearic acid was unaffected (Fig. 2C, *p* > 0.05) and that of palmitic acid was decreased (Fig. 2A, *p* < 0.05) in response to the mitochondrial uncoupler. This result is consistent with our hypothesis that residual mitochondrial electron transport chain activity inhibits the synthesis of unsaturated fatty acids.

## Discussion

The interactions of the multiple pathways of oxygen utilization during alcoholic fermentation are poorly understood, especially in the case of sake brewing, where oxygen-derived substances from rice and koji coexist with sake yeast. The present study is the first to report that sake yeast is the main source of unsaturated fatty acids in fermentation mash, and that unsaturated fatty acid content of the fermentation mash is affected by mitochondrial activity of sake yeast. This novel insight suggests that the content of unsaturated fatty acid in the fermentation mash may be decreased by augmenting the mitochondrial activity of yeast during alcoholic fermentation.

Although shrinkage of mitochondria during anaerobiosis has been reported (Plattner & Schatz, 1969), the time scale of this shrinkage and its effect of other metabolic processes during alcoholic fermentation remain obscure. Our research group has previously shown that mitochondrial activity during sake brewing affects the brewing characteristics, such as malate and pyruvate production efficiencies and carbon flux, of sake yeast (Oba et al., 2014; Shiroma et al., 2014; Agrimi et al., 2014; Kitagaki & Takagi, 2014; Motomura, Horie & Kitagaki, 2012; Horie et al., 2010; Kitagaki, 2009; Kitagaki et al., 2008). However, the role of mitochondria of sake yeast on fatty acid desaturation has not been elucidated to date. The present study is the first to show that, in contrast to the rapid depletion of oxygen during alcoholic fermentation, as indicated by resarulin staining, mitochondrial transmembrane electron potential, as revealed by mitochondrial morphology, is maintained for a certain period of time. Additionally, we demonstrate that the residual mitochondrial activity decreases the content of unsaturated fatty acid of sake yeast. Our findings suggest that the utilization of sake yeasts exhibiting decreased mitochondrial activity should result in increased levels of unsaturated fatty acids during fermentation, thereby reducing estery flavors. This hypothesis requires further verification.

The utilization of oxygen involves pathways other than the mitochondrial transport chain and fatty acid desaturation. The stoichiometric relationship between these two mechanisms reported in this study suggests that they represent one of the main pathways of oxygen utilization; however, synthesis of ergosterol requires 9 moles of O_2_ whereas desaturation of fatty acid requires only 1 mole (Espenshade & Hughes, 2007; Bloch, 1969) and inhibition of mitochondrial activity also affected sterol synthesis (Adams & Parks, 1969). Moreover, several steps of the synthesis of heme inside the mitochondria also requires oxygen. For example, during the synthesis of heme, coproporphyrinogen III reenters the mitochondrion, where the oxygenase converts it to protoporphyrin IX (Protoporphyrinogen IX oxidase (EC 1.3.3.4) using molecular oxygen (Maines, 1988). Therefore, it is considered that mitochondrial electron transport chain, mitochondrial alternative respiration pathway, ergosterol synthesis, heme synthesis and fatty acid desaturation compete for the residual oxygen during alcoholic fermentation, although the quantitative ratio among these pathways seems to depend on the conditions (Salmon, Fornairon & Barre, 1998).

In conclusion, we demonstrate, for the first time, that residual mitochondrial electron transmembrane potential decreases the synthesis of unsaturated fatty acid within endoplasmic reticulum. This novel finding should provide valuable insights into metabolic regulation and engineering of brewery yeasts.

## Supplemental Information

10.7717/peerj.1552/supp-1 Supplemental Information 1 Yeast Summary Raw Data.

## Additional Information and Declarations

### Data Deposition

The following information was supplied regarding data availability: Raw data is available in the Supplemental Information.
